# Online Monitoring of Chip-Based Microscale Perfusion Fermentations

**DOI:** 10.1021/acsomega.5c06552

**Published:** 2025-10-01

**Authors:** Sabrina M. Cramer, Shubham Gurav, David Glinsner, Sven Kochmann, Diethard Mattanovich, Stephan Hann, Tim Causon

**Affiliations:** † 27270 acib - austrian centre of industrial biotechnology, Muthgasse 11, Vienna 1190, Austria; ‡ BOKU University, Institute of Analytical Chemistry, Department of Natural Sciences and Sustainable Resources, Muthgasse 18, Vienna 1190, Austria; § BOKU University, Institute of Microbiology and Microbial Biotechnology, Department of Biotechnology and Food Science, Muthgasse 18, Vienna 1190, Austria

## Abstract

As part of established biomanufacturing development, screening and early phase bioprocess development occurs at bench scale (microplates and shake flasks) whereby conventional offline sampling can only provide limited feedback on fermentation bioprocess parameters including strain productivity. To address these limitations, a new sensitive and selective online analytical platform consisting entirely of commercially available components with a small footprint (valves, 2DLC hardware, LC separation, and online tandem mass spectrometry) was developed for online monitoring of chip-based microbioreactors. Fermentations of microbial cell factories (*Saccharomyces cerevisiae*) were cultivated in 20 μL bioreactors, requiring perfusion of cell culture media at low μL/min rates delivered by syringe pump modules, operated in a multiplexed configuration with a flow-through stream selection valve, and monitored with a 2DLC-MS/MS system adapted for microscale operation. This allows uninterrupted multiplexed microperfusions to be monitored with online measurements of metabolites from parallel fermentations without the occurrence of blockages or cross-contamination between independent fermentations. Fermentations of lactic-acid-producing strains of*S. cerevisiae*were continuously monitored over 5–24 h, demonstrating the suitability of the platform for online monitoring of product quantity and key metabolites for fermentation biotechnology. Offering minimal consumption of biological material and using <1.5 mL of cell culture media over 24 h per experiment, this new platform can be used for monitoring a broad range of biomolecules, rapid strain selection, and screening of microenvironmental factors and is adaptable for targeting other key biotechnology products.

## Graphical abstract

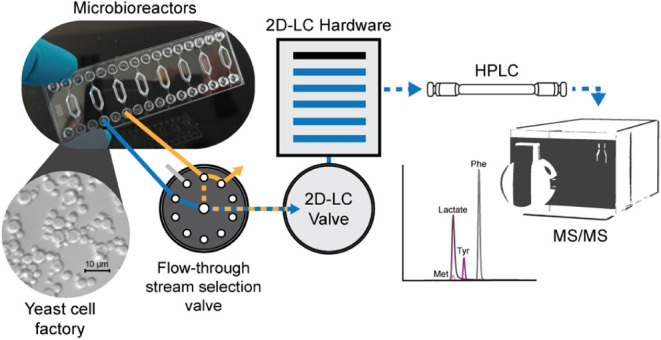

## Introduction

A global increase in demand for biological therapeutics such as those in vaccine production, novel drug therapies, and personalized medicine have driven a need for more efficient bioprocess development.^1,2^ Bottlenecks in biopharmaceutical manufacturing can result as the scale of demand outstrips capacity. Similarly, industrial bioproduction of chemicals requires efficient strains that convert substrates fast and with high yield into the desired products.^3^ With finite resources available, bioproduction technologies used for these goals must adapt. To improve production efficiency, microbial strains and cell line clones with higher productivity and long-term stability need to be selected.^4^ Current bioprocess development begins with optimizing conditions and screening for the best producing strains and clones selected at bench scale before ramping up to production level.^5,6^ However, this process is expensive, laborious, and time-consuming as only a limited number of factors can be examined simultaneously. To this end, microfluidics with high-throughput screening capabilities on a downscaled setup are being explored to overcome these limitations.^1,7,8^ This resource-light approach is especially compatible with perfusion fermentations that deliver higher volumetric productivity than fed-batch processes and a more consistent product quality.^9,10^

Previous work with yeast cell factories has demonstrated the value of microscale format for reproducing microbial shake flask cultivations of small molecule and recombinant protein products.^9,11^ A microbioreactor prototype with integrated sensors was developed to rapidly cultivate and screen multiple yeast strains in perfusion fermentations.^9^ These microbioreactor cultures were comparable to shake flasks and the best producer by titer could be identified up to four times faster with the benefits of reduced reagent consumption and culture volume, albeit with off-line analytics for determination of product concentration.

For analytical methods currently applied to fermentation biotechnology, most measurements are typically performed off-line or at-line rather than online and do not allow for continuous measurements to be performed.^7,12,13^ In cases where it is feasible, online sampling carries some significant advantages including lower contamination risk from minimized sample manipulation, real-time analysis reducing unwanted degradation of metabolites before measurement, reduction of waste, and the possibility of automatic process control.^14,15^ For optimal monitoring of bioprocesses, key quantitative data must be concurrently collected to monitor product quality and to track intermediate metabolites as well as side- and end-products resulting from the fermentation processes.^14^ For measuring intermediates, byproduct and product small molecules (metabolites) from biotechnology bioprocesses, liquid chromatography–mass spectrometry (LC-MS) is the gold standard.^16^ LC-MS is typically performed offline for biotechnology applications meaning that compatibility with microscale formats is not straightforward.^17^ Some recent work coupling MS to microfluidic systems for cellular level measurements has demonstrated how the benefits of both microscale systems and LC-MS can be realized on a single platform. For example, heart-cutting 2DLC methods provide a means to store multiple sample fractions in loops for subsequent analysis with different techniques. This approach was applied previously to monitor drug metabolism over time, by coupling organ-on-a-chip liver organoids to LC-MS with a 2-port, 10-position valve using two sampling loops to collect and analyze cell media effluent at low flow keeping the system pressures low and compatible with organoids.^18^ In another recent study, perfusate from human islets held in a glass microfluidic device was sampled every 2 min to measure changes in cellular secretion using a 6-port valve to store samples collected at 1 μL/min before LC-MS/MS analysis.^19^

In this regard, the integration of low-volume microfluidic cell factories with analytical LC-MS in an online fashion would present a new and valuable platform for the development and optimization demands of fermentation biotechnology. Despite the benefits that online sampling directly from microfermentations for LC-MS analysis might have, the development of such a platform presents several challenges. An ideal platform needs to incorporate many aspects, including uninterrupted perfusion in all microchip chambers, efficient separation, sensitive and selective MS detection, as well as suitable hardware, connectors, and software to manage microperfusion sampling on a routine basis. Preferably, the platform should also comprise of readily available commercial products and allow for multiplexed monitoring of multiple perfusion fermentations of industrially relevant microbial host strains in parallel. Thus, to address these challenges, we developed a sensitive and selective analytical platform comprising of valves, 2DLC hardware, with online LC separation and tandem mass spectrometry using only commercially available components.

## Experimental Section

### Chemicals

Working solutions and eluents were prepared with ultrapure water supplied by a Milli-Q IQ 7000 water system equipped with a Bio-Pak polisher cartridge from Merck Chemicals and Life Science GmbH (Vienna, Austria). LC-MS grade methanol, ethanol (EtOH), and formic acid were acquired from Sigma-Aldrich (Vienna, Austria). l-phenylalanine and *trans*-aconitic acid were from Sigma-Aldrich. For medium preparation, ethanol 96% was from Merck Millipore (Germany), urea and D­(+)-glucose monohydrate from Carl Roth GmbH + Co. KG (Germany), and yeast nitrogen base without amino acids and ammonium sulfate from Becton, Dickinson and Company (France). For pH control in the microbioreactor, citric acid and trisodium citrate dihydrate (Carl Roth GmbH, Germany) were used to prepare buffered medium. All chemicals used had purities greater than 99% unless otherwise stated.

### Yeast Strains and Cultivation Conditions Used in This Work

All strains are based on*Saccharomyces cerevisiae*in-house yeast strains producing L-lactic acid by overexpression of *Lactobacillus plantarum* lactate dehydrogenase. As previously described by Totaro et al.^8^ a low producing strain (1a) and a high producing strain (1e) were used.

For biomass formation, YNB + E medium containing 10 g/L ethanol, 4.54 g/L urea, and 3.4 g/L yeast nitrogen base (YNB) was used. YNB + G medium containing 5 g/L ethanol, 4.54 g/L urea, 3.4 g/L YNB, and 200 g/L glucose was used for lactic acid bioconversion.

The cells cultivated on Petri dishes were transferred into 10 mL YNB + E medium and incubated at 30 °C, 180 min^–1^ and a relative humidity of 70%. A 1-to-10 volume ratio was kept constant for every preculture. On the fourth day, cells were centrifuged (2,000 g, 10 min, 20 °C) and resuspended in YNB + G at a biomass concentration of OD600 = 40 for lactate production experiments. The OD was measured by a Biochrom WPACO8000 Cell Density Meter.

Cells were loaded into 20 μL hydrophilized reaction chamber Topas microchips from microfluidic ChipShop (Jena, Germany). The microchips were cleaned the day before use by triple rinsing with water and 70% (v/v) EtOH and allowed to dry. Immediately prior to use, the chambers were flushed once with 70% EtOH and then triply with YNB + G medium. Durapore membrane filters (0.45 μm, hydrophilic PVDF) from Merck Chemicals and Life Science GmbH (Vienna, Austria) were punched out to size, rinsed with 70% EtOH before use, dried, and placed in the outlets of the microchip. During inoculation, the 20 μL microchip chambers were filled with the OD40 cell suspension by pipetting. Excess YNB + G medium was pipetted into the inlet and outlet ports of the chip to remove the potential for air bubbles when placing the chip in the incubator.

### Instrumentation

The primary 2DLC hardware comprised of a 1290 Infinity II 2D-LC System from Agilent Technologies (Waldbronn, Germany), equipped with one 1290 high-pressure binary pump and one 1260 quaternary pump, and a Multicolumn Thermostat set at 30 °C. The ^1^D detector was removed from the system and the ^1^D pump replaced with an external Harvard Apparatus Pump Controller (Holliston, MA, USA) connected to three Pump 11 Pico Plus Elite dual syringe pump modules. An Agilent Ultivo triple quadrupole mass spectrometer (Agilent Technologies, Singapore) equipped with a Jetstream ESI source was used for online ^2^D detection.

The first and second dimensions were interfaced by a 10-port/4-position active solvent modulation (ASM) valve (configured in countercurrent mode) connected via two 1.9 μL MP35N transfer capillaries (170 × 0.12 mm) to two 14-port/2-position deck valves carrying six stainless steel sample loops with a volume of 40 μL each (420 × 0.35 mm) or six MP35N 10 μL loops (104 × 0.35 mm). The standard pressure release kit installed between the ^1^D detector and the ASM valve was removed.

A 10-port, 5-position flow-through stream selector (FSS) valve from VICI Valco Instruments with 1/32” PEEK fittings, 0.25 mm bore size, and wetted parts made of biocompatible materials (PAEK stator and Valcon E rotor) was controlled through a high-speed universal electric actuator. The actuator was connected to an Agilent Infinity 1200 Universal Interface Box II with an external contact cable (Agilent General Purpose Cable GPIO-Open End) and automatically controlled by contact closure in ChemStation (Table S10).

Two computers, one with Agilent MassHunter (Ultivo LC/TQ 1.2 (Build 1.2.23)) installed and the other with OpenLab CDS ChemStation (Rev. C.01.10 [314]) software and Agilent 1290 Infinity 2D-LC software add-on (version A.01.43) installed, were used to operate the system, control the valves, and acquire all LC-MS data.

### Microchip Interface

The microchips were placed into a Lab-on-a-Chip Cell Culture Incubator from microfluidic ChipShop with a temperature control unit set to 37 °C. For the on-chip perfusion cultures, the inlet ports of the incubator were connected through PEEK tubing (1/32” outer diameter, 63.5 μm inner diameter) to 1 mL Omnifix F Luer Lock Solo plastic syringes from B. Braun (Melsungen, Germany) filled with YNB + G medium placed in the infusion pump modules. The YNB + G medium was delivered to fermentations in microchips at flow rates of 1 μL/min or less. The outlets of the incubator were connected to the FSS valve with 1/32” PEEK tubing through the directly integrated fluidic interfaces on the incubator. An additional Pico Plus Elite syringe pump module was used for washing the sample loops in between fractions collected from different fermentations with water and another for flushing the system in between analyses. A wash solution of 10 μmol/L *trans*-aconitate was used between independent fermentations as an internal standard to ensure the stability of the platform remained consistent throughout the duration of the fermentations.

### LC-MS/MS and 2DLC Settings for Monitoring Online Microperfusion Fermentations

Separations were performed with an ACQUITY Premier HSS T3 RPLC column (2.1 × 50 mm; 1.8 μm *d*_*p*_) equipped with a VanGuard FIT column from Waters (Wexford, Ireland) at a temperature of 30 °C. The following gradient program was used with eluent A (99.9% v/v water, 0.1% v/v formic acid) and eluent B (100% v/v methanol) at a flow rate of 0.1 mL/min. See Table S1 for the gradient program. As active solvent modulation was not used, a 4 min gradient time and 4 min postgradient time resulted in a ^2^D-cycle time of 8 min. All 2DLC method parameters are listed in Table S4. Source conditions for MS detection are listed in Table S2 and all MS/MS transitions are listed in Table S3. A fully detailed schematic of the platform is shown in Figure S3.

### Data Analysis

Data analysis was performed in Agilent MassHunter Workstation Qualitative Analysis and Quantitative Analysis (11.1) and Microsoft Excel. R Studio (2024.12.1 Build 563) was used for statistical analysis.

## Results and Discussion

In perfusion fermentations, cell media is continuously replenished, supplying a steady source of nutrients, while waste medium is removed from the culture. Microfluidic perfusion fermentations have previously been shown to increase strain productivity in*S. cerevisiae*strains, with the lactic acid production rate rising with perfusion rate, as well as delivering results on a faster time scale compared to batch cultures.^9^ Microscale perfusion fermentations also offer better compatibly with online sampling than with batch fermentations as continuous flow conditions can be maintained. To provide this constant supply of fresh cell medium for a system with microscale bioreactors of 20 μL, a small-footprint pump controller with dual syringe pump modules was implemented. This carries several advantages: its flow performance at low perfusion flow rates, the possibility to control multiple pumps from a single control module, and the compatibility with sterility requirements for fermentations. Moreover, this setup also allows the total amount of media consumed to be kept even lower via the use of low-volume, disposable syringes.

To assess the tolerance of the ESI-MS/MS system toward the cell media used in microbial perfusion fermentations, fractions of extracellular effluent from shake-flask cultivations were collected and measured online with and without a LC separation. As expected, the addition of an LC column reduced ion suppression and provided stable retention times for confirming metabolite identity in data processing, and was thus used in all further experiments (Figure S4).

### Enabling Compatibility of Microperfusion Flow Regime with Analytical Platform

Adapting an analytical LC-MS platform to be fully compatible with microscale fermentations posed some additional technical challenges primarily due to the low perfusion flow rates of only a few μL/min or less. Thus, a balance between the viability of microperfusion conditions and stable performance of the ESI source flow requirements of several tens of μL/min (minimum) must be sought. The required flow rates for microperfusion for microbial fermentations using*S. cerevisiae*can be readily estimated from previous work highlighting the impact of perfusion rate (*P*) on lactic acid production, with a maximum growth rate achieved at *P* = 1 h^–1^ in 15 μL microbioreactors.^9^ This perfusion rate is equivalent to a flow rate of 333 nL/min for a 20 μL microbioreactor. However, working at this flow rate would greatly increase the time needed to perform an analysis on this platform, with each collected fraction requiring 24 min on the standard installed 2DLC hardware (Figure 1). Accordingly, a perfusion rate of *P* = 3 h^–1^ was selected as a compromise, reducing the time required to collect one cut to 8 min on the standard platform (ideal for online LC-MS), but still maintaining representative perfusion conditions for yeast based on previous work.^9^

**Figure 1:**
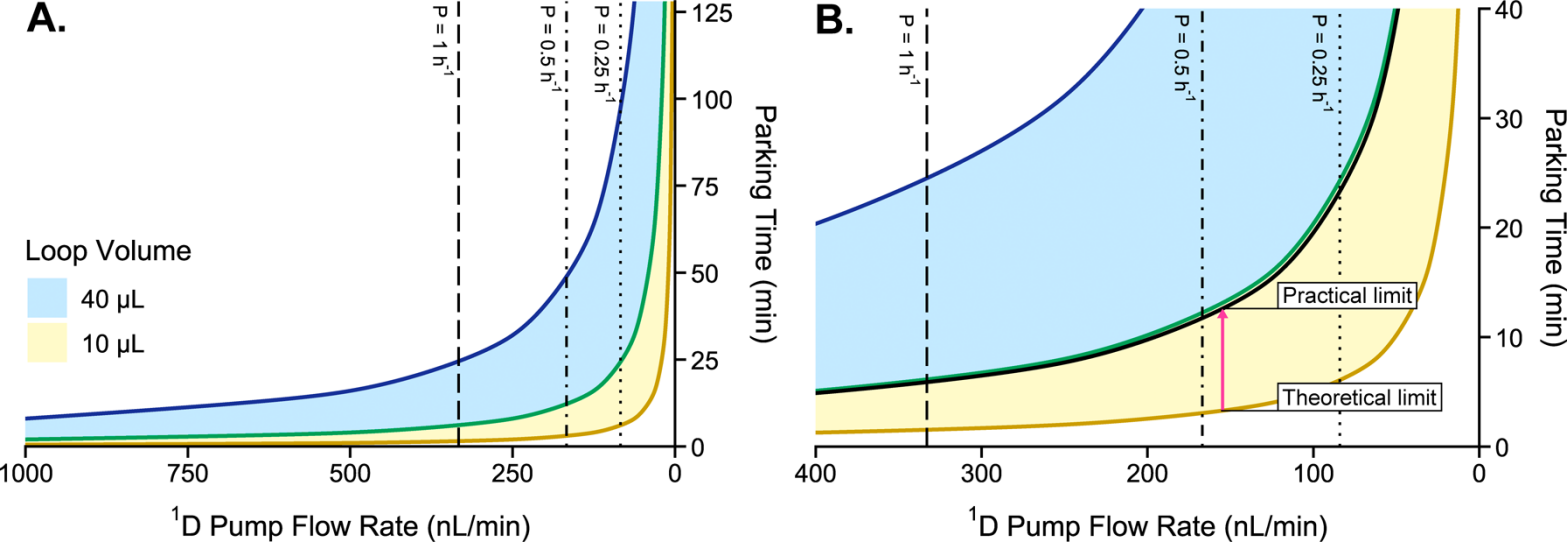
Representation of the sampling time required to park one cut (i.e., collect one fraction in a sample loop) for (A) the range of 5–20% sample loop filling according to microperfusion flow rate delivered by the 1D pump. Both 40 μL (
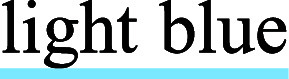
) and 10 μL sample loops (
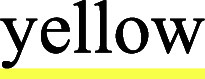
) were considered. The upper boundaries represent 20% loop filling (
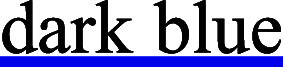
 for 40 μL loops and 
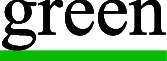
 for 10 μL loops) and the lower boundaries are for 5% loop filling (
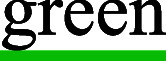
 for 40 μL loops and 
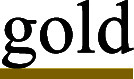
 for 10 μL loops). Typical microfluidic perfusion rates of *P* = 1, 0.5, 0.25 h^–1^ are indicated by dashed lines. (B) Zoomed in region of relevant perfusion rates for the present work. The 
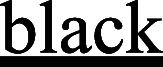
 line represents the practical limit imposed on the system due to the contribution of transfer capillary volume.

### Necessary Modifications to Standard 2DLC Hardware and Operation

In conventional 2DLC operation, the transfer of the effluent from the first dimension to the second dimension occurs via the sample loops on the deck valves. There are six loops on each deck valve into which the ^1^D effluent flows during a set sampling time that determines the volume of each collected fraction. In the present work, each of these cuts represents a snapshot of the perfusion fermentation integrated across the sampling time. For the analysis, the 2DLC was operated in high-resolution sampling mode in which the valve switches directly before and after each cut allowing for precise fractions to be collected. A maximum five back-to-back cuts can be taken per deck valve as the first loop is used as a bypass during flow. Successive fractions over the duration of the fermentation are collected in the sample loops and transferred (injected) onto the LC column which is serviced by the higher flow rate of the ^2^D pump upon a valve switch (Figure 2). To reduce carryover, the valve was operated in countercurrent mode where the fraction is collected in one flow direction and then displaced from the capillary in the opposite direction to which it was collected. After each measurement, the sample loops and transfer capillary are automatically flushed by the 2DLC software while uninterrupted perfusion fermentations continue in the microchip chambers. Lastly, a syringe pump module was used to flush the path from the FSS valve to the deck valve with water (10 μL/min), removing traces of the previous sample.

**Figure 2:**
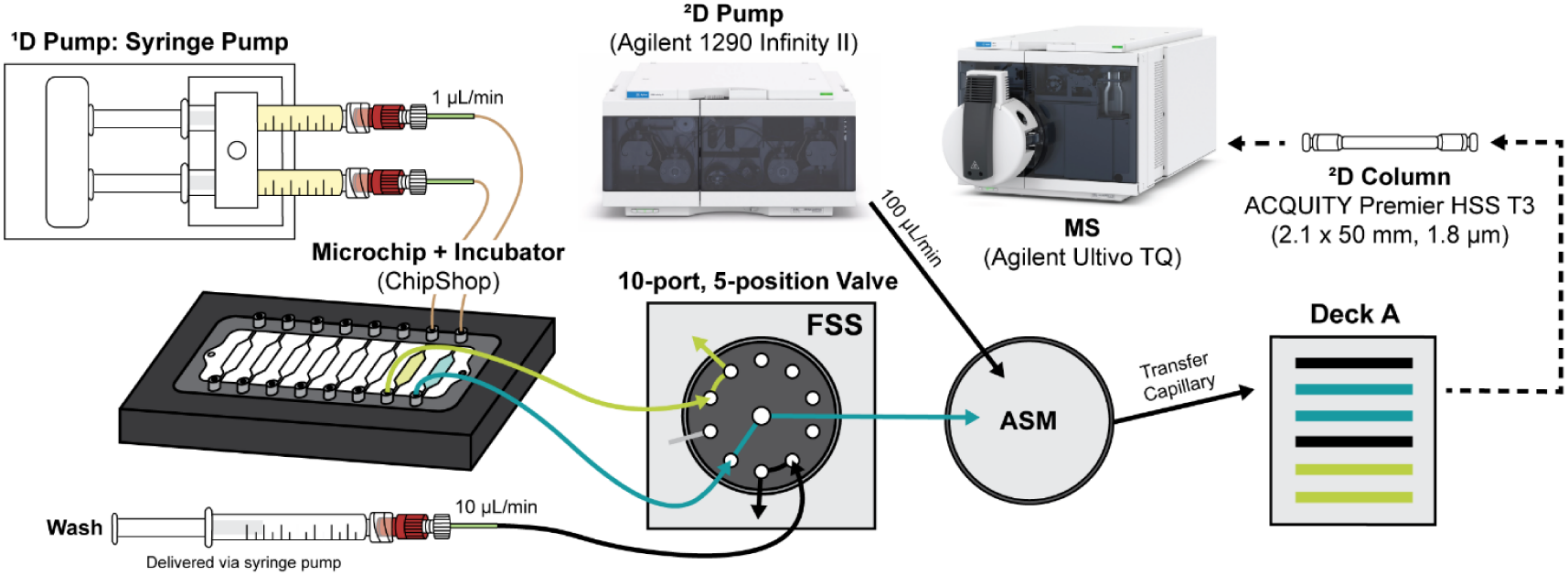
Schematic representation of the full 2DLC-MS/MS analytical μ-platform. The multiplexed perfusion fermentations in microbioreactors are performed at 37 °C inside a microchip incubator with a syringe pump delivering cell medium at 1 μL/min. An additional syringe pump is used to deliver the wash flow at 10 μL/min to flush the flow path after each measurement. The 10-port, 5-position flow-through stream selection (FSS) valve is used to switch between independent fermentations and wash without interruption of flow in any microchip chambers. The 2DLC hardware (ASM valve and Deck A sampling loops) is used to collect sequential fractions from each perfusion fermentation being monitored. Collected fractions are transferred onto the LC column from the sample loop on Deck A using a 100 μL/min flow rate from the ^2^D pump and analyzed by MS/MS. The effluent from fermentations not actively being transferred to the ASM valve (
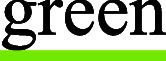
) can be transferred to other analytical modules or discarded to waste. See Figure S3 for detailed schematics of all valve operations. (Instrumentation images reproduced with permission courtesy of Agilent Technologies, Inc.).

Due to the increased time constraints imposed upon the system via the lower absolute flow rates needed for maintaining fermentation viability, several parameters were investigated for optimization including the filling percentage of the sample loops used to collect the fractions, the volume of the sample loops on the deck valves, and the timing of valve switches.

In contrast to traditional 2DLC, the slow microperfusion flow rates employed in this work greatly increase the time required to collect one cut. The minimum sampling time is limited by the volume of the transfer capillary installed between the ASM valve and deck valve. This time is automatically determined by the software through the 2DLC configuration settings and the ^1^D pump flow rate. To this end, the quaternary pump was used as a ^1^D pump solely for software compliance purposes and its flow rate set to the pump minimum of 1 μL/min with flow directed to waste.

As a minimum of 20% loop filling is recommended by the manufacturer, the standard 40 μL sample loops on the deck valves were replaced with 10 μL sample loops to reduce the time per cut (Table S9). The sampling time *t*_*s*_ was calculated according to eq 1:

1 $$t_{s}=\frac{f\cdot V_{l}}{F_{1}}$$

where f is the fraction of loop filling, *F*_1_ is the ^1^D flow rate, and *V*_1_ is the sample loop volume.

However, the volume of the installed transfer loop capillary practically limits the theoretical gains from switching to lower volume sample loops as the last cut sampled is stored in the transfer capillary (Figure 1B). To address this experimentally, three different sample loop filling percentages were tested: 20%, 10%, and 5%. This was achieved by lowering the ^1^D pump module flow rate and collecting four cuts of a solution containing 8 μmol/L of phenylalanine. A 20% loop filling of the 10-μL sample loop was obtained by taking one cut of 2 min at a flow rate of 1 μL/min, a 10% loop filling with a flow rate of 500 nL/min, and a 5% loop filling with a flow rate of 250 nL/min. Finally, a sample loop filling percentage of 20% with 10-μL loops was chosen for all subsequent experiments as it yielded the lowest RSD (3.9%) across four cuts (Figure S5).

For standard 2DLC instrumentation, the transfer capillary between the ASM valve and deck valves represents the entirety of the path volume and corresponds to the minimum sampling time required to stop contamination from one cut to another. However, the aim in this work of assessing multiplexed fermentations required alteration of the flow path for using the syringe pump and FSS valve. The increased path volume between the FSS and ASM valve required a corresponding adjustment to the minimum sampling time applied. As the software automatically determines this value, an additional sampling delay time to allow for complete filling of the flow path was calculated and experimentally confirmed. This delay time was entered into the sampling table as the start time in the 2DLC method (Table S5).

The volumes in the system along the transfer path are much more relevant when the perfusion flow rate is ≤1 μL/min compared to the high flow rates normally used with these 2DLC valves. Considering the volume of the transfer capillary (1.92 μL, 170 × 0.12 mm) and the capillary added between the FSS and ASM valve (0.60 μL, 190 × 0.0635 mm), a theoretical total volume of 2.52 μL was expected (Table S8). However, when using a perfusion flow rate of 1 μL/min, an additional sampling delay time of 0.6 min was found to be inadequate to prevent carryover. The true path volume was determined experimentally by taking three cuts and prefilling the total path with a blank or a sample. The sampling delay time needed to be increased by 0.38 min to reduce the RSD across the peak area of the cuts and reduce the signal (peak area) seen when flushing the capillaries at the end of every measurement. This resulted in a practical total path volume of 2.90 μL, with the additional 0.38 μL likely occurring from unaccounted for volume inside the valves that is only relevant at such low flow rates (Figure S6).

Additionally, the timing of the switches of the FSS valve needed to be set so that the sampling delay time allows the cuts to be preloaded into the capillaries while other cuts are being collected (Figure S7). The total analysis time for one collection and corresponding measurement is the sum of the analysis times of each cut and the time needed to flush the capillaries in the decks (which is automatically programmed by the software and equal to the time of one ^2^D-cycle), plus the sampling delay time. The resulting timing tables are shown in Tables S6 and S7.

Despite taking these steps, the carryover between independent perfusion fermentations was not eliminated when setting the sampling delay time to 0.98 min (with the automatic delay of 1.92 min implemented by the software). This contamination cannot be analytically corrected for without increasing the complexity of the system and thus decreasing robustness, so a washing step was incorporated between independent fermentations to flush the path between the FSS and deck valve. As the wash flow rate must match the ^1^D flow rate of the first pump module to avoid washing the samples out of the sampling loops, a third pump module operating at 1 μL/min was used for this purpose. This allows fractions from microbioreactors operating in parallel to be infused at low perfusion flows, collected in sampling loops, transferred to the LC-MS/MS for measurement with washing steps to preventing cross-contamination. It is noted that stepping backward with the FSS valve is not recommended as the transition through the different streams will introduce sources of contamination. Thus, for use with the currently employed FSS valve, five cuts are collected per time point as follows: two cuts of the first fermentation, the wash, and then two cuts of the second fermentation (see Figure S8).

### Application of Platform to Multiplexed Monitoring of Lactate-Producing Yeast Strains

To demonstrate the capabilities of the full system for its intended use, both single and multiplexed microscale fermentations of lactic-acid-producing strains of*S. cerevisiae*were performed in 20 μL bioreactors focusing on online monitoring of the product and other metabolites. For all experiments, the engineered lactic-acid-producing strains were cultivated in the microfluidic device, where a continuous 1 μL/min flow of cell medium was pumped through the 20 μL cultivation chambers within which cells were retained by placing a 0.45 μm PVDF filter in the outlet ports before inoculation (Figure S1). The addition of a filter was found to be necessary for ensuring cell retention within the microchip chambers during perfusion operation (Figure S2).

To demonstrate the long-term stability of the system at time scales of relevance for fermentation biotechnology (i.e., up to 24 h), the production of lactate was initially monitored in a single fermentation of the high-producing strain 1e. Lactate was detected in all successive cuts over a 5 h fermentation and revealed that the production quickly stabilized after approximately 2 h of monitoring with the peak area of lactate remaining consistent thereafter with 4.0% RSD determined across three cuts at the 5 h mark (Figure S8A). Furthermore, lactate could still be reliably detected after 24 h of continuous operation without any disruptions to the system (e.g., blockages), demonstrating the stability of the entire system for typical fermentation time scales in biotechnology. With the FSS valve used in the current setup, such experimental comparisons are possible for multiplexed monitoring of a maximum of five different flowing streams. With one flow-through stream dedicated to the postmeasurement wash flow, and one other stream for intermediate washing between independent fermentations, a total of three perfusion experiments can be monitored. The stable performance observed in single-fermentation assessments could also be demonstrated for monitoring of lactate in two multiplexed fermentations performed in parallel chambers on a single microchip.

To further demonstrate the capability of the platform for measuring additional extracellular metabolites covered by analytical LC-MS/MS, the panel of monitored metabolites was expanded to include phenylalanine, isoleucine/leucine, tyrosine, and methionine (Figure S10). These cellular metabolites are routinely quantified for yeast biotechnology metabolomics with RPLC-MS and are relevant for the strains of lactate-producing yeast considered in this work as indicators of bioprocess stability under perfusion growth conditions. The peak area repeatability of the entire panel was assessed for biological replicates. Initially, two biological replicates of the 1e strain were monitored concurrently across an 8 h fermentation time. The measured lactate peak area remained within expected biological variation after allowing 2 h for stabilization, with 9.1% to 11% RSD determined across four cuts per time point.

Additionally, these metabolites were also monitored in multiplexed fermentations with the 1e and 1a strain over 5 h. As expected, the fermentations quickly reached a ready state, with greater production of lactate in the fermentation with the 1e strain than with the 1a strain observed (Figure 3). After initial rapid changes in metabolite abundances in the first 2 h, the abundances of product (lactate) and amino acids reach steady levels that are maintained across several hours of lactate production, which is in good agreement with previous results.^9^ An RSD of 2.9% for the internal standard (*trans*-aconitate) peak areas from the wash sampling cuts between the independent fermentations indicated that the platform remained stable during the entire 5 h fermentation, which is critical for robust comparison of different strains assessed within a single experiment. All other metabolites were found to be detected in similar concentrations across both strains and with much higher concentrations than present in the cell media background (Figure S9).

**Figure 3:**
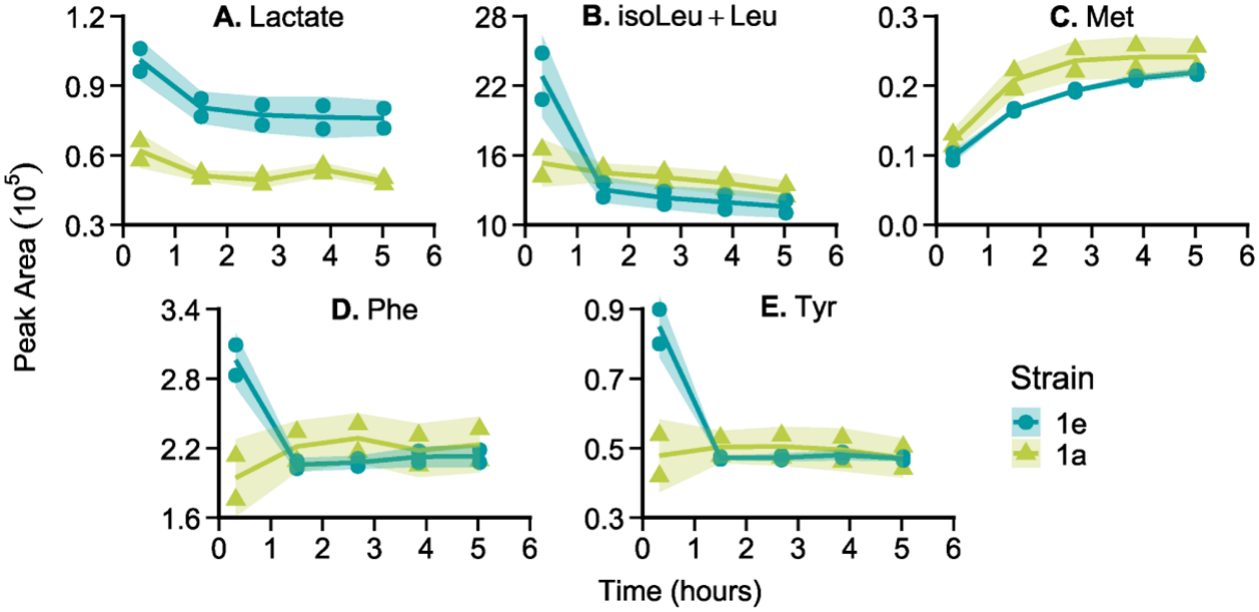
Results for metabolites monitored over 5 h fermentations of*S. cerevisiae.* The peak area for each metabolite was measured every hour for lactate (A), isoleucine/leucine (B), methionine (C), phenylalanine (D), and tyrosine (E) during concurrent 20 μL perfusion fermentations with 1a (▲), the*S. cerevisiae*strain with lower production of lactate, and 1e (●), the higher-producing strain. For each time point, both fermentations were measured in duplicate. The shaded areas represent the standard deviation of the duplicates using a correction factor to compensate for the small sample size, as proposed by Roesslein et al.^20^.

Finally, to test the potential of this platform as an effective tool for rapid screening of different fermentation conditions and strains, two different media conditions were examined for growth of*S. cerevisiae*strains. The standard medium used has an initial pH of 4.3, but as lactic acid accumulates in the culture medium, the pH lowers, decreasing cell metabolism and the production of lactic acid by the cells. Citrate-buffered media was prepared at pH 3 and 5, to monitor the differences in production under a harsh and beneficial environment. The buffering capacity of the media during the fermentation was monitored via pH test strips by testing the flow-through effluent from the FSS valve outlet ports. Five cuts were collected and analyzed online over 8 h fermentations for the four conditions assessed (i.e., strains 1e and 1a with fermentation media buffered at pH 3 and pH 5). As expected, since a pH 5 environment is less stressful to the cells than pH 3, both strains struggled more at the lower pH and produced significantly less lactate at pH 3 than at pH 5 (*p* < 0.0001, Figure 4). These results are in-line with previous results reported with*S. cerevisiae*microbioreactor fermentations^9^ and demonstrate that the new platform can monitor different strains and bioprocess conditions at relevant time scales and with minimal consumption of biomass and reagents.

**Figure 4:**
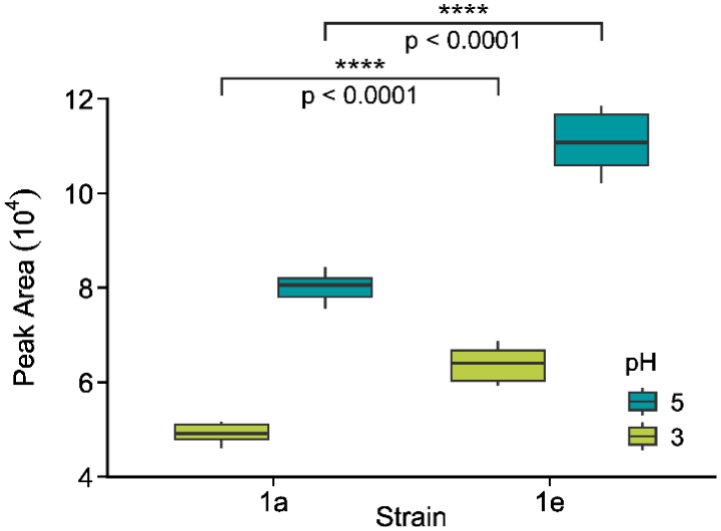
Comparison of lactate production in the 1a (low-producing) and 1e (high-producing) strains at different pH. The cell media was buffered at pH 3 or 5 for the 8 h perfusion fermentations with an analysis rate of five sequential cuts every hour. Lactate peak area for each strain was compared across 5 h (two biological replicates per strain measured at 4 time points during the fermentation) at pH 3 and 5 after stabilization in lactate production. Statistical analysis comparing the two strains at pH 3 and 5 was performed with a two-sided Welch Two Sample *t* test with unequal variance. **** represents a significance level of *p* < 0.0001.

## Conclusion

An analytical platform for online monitoring of multiplexed fermentations in 20 μL microbioreactor that combines 2DLC hardware, valves and LC separation with MS detection was developed and adapted for microperfusion flow rates. The platform was successfully employed for continuous monitoring of lactate production in multiplexed fermentations of*S. cerevisiae*strains for up to 24 h under different bioprocess conditions with online MS/MS analysis of products and metabolites. Differences between producer strains and their performance under various conditions can be ascertained on a short-time scale and with minimal consumption of biological material and cell media.

In the context of fermentation biotechnology, this new platform has demonstrated capability for more efficient strain selection, with a reduction in required time and expense by allowing various bioprocess parameters to be screened in an online manner. Although not shown in this work, we expect that other key biotechnology products such as recombinant proteins or antibodies can also be addressed with this platform. Finally, the current operating capacity of the platform can be further expanded with improved valve designs or the addition of sensors to extend the range of available time-correlated data and applications (e.g., stable-isotope-label tracing that could be induced via deliberate perturbations of individual perfusion cultivations).

## Supplementary Material



## Notes

Details of experimental methods and results; tables of MS/MS transitions, LC gradients, and valve switch timing; figures of the microfluidic chip and connections, analytical platform components, and valve hardware (PDF)
