# Supplementary material for: Online Monitoring of Chip-Based Microscale Perfusion Fermentations
Source: ACS Omega. 2025 Oct 1;10(40):47355–62. doi: 10.1021/acsomega.5c06552 (PMC12529116; doi:10.1021/acsomega.5c06552)
Supplement: Supplementary file 1 [file ao5c06552_si_001.pdf]

Supporting Information

Online monitoring of chip-based microscale perfusion fermentations

Sabrina M. Cramer[1,2], Shubham Gurav[1,3], David Glinsner[1,3], Sven Kochmann[1,2], Diethard Mattanovich[1,3], Stephan Hann[1,2], Tim Causon[1,2*]

1. acib - Austrian Centre of Industrial Biotechnology, Muthgasse 11, 1190 Vienna, Austria

2. BOKU University, Institute of Analytical Chemistry, Department of Natural Sciences and Sustainable Resources, Muthgasse 18, 1190 Vienna, Austria

3. BOKU University, Institute of Microbiology and Microbial Biotechnology, Department of Biotechnology and Food Science, Muthgasse 18, 1190 Vienna, Austria

*Corresponding author: tim.causon@boku.ac.at

Table of Contents

S1 Analytical method information....4
S1.1. LC-MS/MS parameters....4
S1.2. 2D-LC-MS/MS parameters....5
S1.3. FSS valve parameters....6
S1.4. Sampling loops....6
S1.5. Data analysis parameters....7
S2 Analytical platform assembly....7
S2.1. Microchip preparation....7
S2.2. Analytical platform components....9
S3 Adapting the platform for microscale operation....10
S3.1. Hardware and setting modifications....10
S3.2. Flow path volumes....13
S4 Monitoring metabolites from microscale fermentations....15
S4.1. Metabolites measured in single and multiplexed fermentations....15

Overview of Tables

Table S1. LC-MS/MS gradient....4
Table S2. LC-MS/MS method parameters....4
Table S3. TQ MS/MS transitions....4
Table S4. 2D-LC method parameters....5
Table S5. 2D-LC sampling table....5
Table S6. 2D-LC sampling and valve switch timing....5
Table S7. FSS valve switch timing....6
Table S8. Flow path volumes.....6
Table S9. Time to park one cut with different sampling loops....6
Table S10. MassHunter Quantitative Analysis MS/MS method parameters....7
Table S11. Components in the multiplexed microchip fermentation interface....10

Overview of Figures

Figure S1. Overview of the steps to prepare the microchip for use 7

Figure S2. Off-line lactate production with and without cell retention filters...................8

Figure S3. A schematic representation of the entire 2DLC-MS/MS platform....................9

Figure S4. Analysis with and without LC separation 11

Figure S5. Comparison of 20%, 10%, and 5% sample loop filling 12

Figure S6. Experimental determination of the optimal sampling delay time 13

Figure S7. Platform operation under optimal timing conditions 14

Figure S8. EICs of lactate in single and multiplexed fermentations................................15

Figure S9. Background level of metabolites in cell media. 16

Figure S10. Representative EICs for monitored metabolites...165

S1 Analytical method information

S1.1. LC-MS/MS parameters

Table S1. LC-MS/MS gradient. A = 0.1% v/v formic acid; B = methanol. [2]D Flow rate was 0.1 mL/min. Total run time was 8 min.

Time (min)	%A	%B
0.00	95	5
2.00	60	40
4.00	0	100

Table S2. LC-MS/MS (TQ) method parameters.

**Ion source**	Dual AJS ESI
**Drying gas temperature**	300 °C
**Drying gas flow**	10 L/min
**Sheath gas temperature**	195 °C
**Sheath gas flow**	3.5 L/min
**Nebulizer**	10 psi
**Capillary voltage**	2000 V

Table S3. TQ MS/MS transitions.

Compound	Precursor ion (*m/z*)	Product ion (*m/z*)	Fragmentor (V)	Collision energy (V)	Polarity
Tyrosine	182.1	136	80	19	Positive
Phenylalanine	166.1	103	88	30	Positive
Methionine	150.1	56	46	20	Positive
Leucine/Isoleucine	132	86	56	10	Positive
Lactate	89	42.9	56	10	Negative

S1.2. 2D-LC-MS/MS parameters

Table S4. 2D-LC method parameters.

**2D-LC mode**	High-resolution sampling
**Valve topology**	Concurrent
**^{2}D gradient stop time**	4 minutes
**^{2}D cycle time**	8 minutes
**^{1}D flow (syringe pump)**	1 µL/min
**^{2}D flow**	100 µL/min
**Delay time**	0.98 min
**Transfer capillary fill time at 1 µL/min**	1.92 min
**Sampling time**	2.90 min
**Number of cuts collected**	5

Table S5. 2D-LC sampling table from the 2D pump setup parameters.

Time (min)	Mode	Sampling time (s)	Cuts
0.98	Time-based	174	5

Table S6. 2D-LC sampling and valve switch timing table. Calculated based on number of cuts, delay time, and sampling time in Table S5.

Time (min)	Event
5.80	Cut 1 parked
5.80	FSS valve switch (P1 to P2)
8.70	Cut 2 parked
8.70	FSS valve switch (P2 to P3)
11.60	Cut 3 parked
14.50	Cut 4 parked
15.48	Cut 5 analysis
23.48	Cut 4 analysis
31.48	Cut 3 analysis
39.48	Cut 2 analysis
47.48	Cut 1 analysis
63.48	LC and MS stop

S1.3. FSS valve parameters

Table S7. FSS valve switch timing table for the UIB.

Time (min)	External contact closure	Valve switch	Description
0.00	A	P5 to P1	Wash to fermentation 1
5.80	B	P1 to P2	Fermentation 1 to wash
8.70	B	P2 to P3	Wash to fermentation 2
55.48	B	P3 to P4	
55.49	B	P4 to P5	Cleaning

Table S8. Flow path volumes.

Path	Length (mm)	ID (mm)	Volume (µL)
FSS valve to ASM	190	0.0635	0.60
Transfer capillary	170	0.12	1.92

S1.4. Sampling loops

Table S9. Time to park one cut with different sampling loop volumes as per Equation 1.

	10 µL sampling loop		40 µL sampling loop	
Flow Rate (nL/min)	5% loop filling (min)	20% loop filling (min)	5% loop filling (min)	20% loop filling (min)
1000	0.5	2	2	8
500	1	4	4	16
250	2	8	8	32
125	4	16	16	64
62.5	8	32	32	128
31.25	16	64	64	256
15.625	32	128	128	512
7.813	64	256	256	1024
3.906	128	512	512	2048

S1.5. Data analysis parameters

Table S10. Example of MassHunter Quantitative Analysis TQ method parameters. Retention times were calculated from cut analysis times (Table S6).

Name	TS	Precursor Ion (m/z)	Product Ion (m/z)	RT (min)	Smoothing	Smoothing Function Width	Smoothing Gaussian Width
lactate_cut3	1	89	42.9	12.3	Gaussian	15	5
lactate_cut2	1	89	42.9	20.3	Gaussian	15	5
lactate_cut1	1	89	42.9	28.3	Gaussian	15	5
lactate_flush	1	89	42.9	36.3	Gaussian	15	5

S2 Analytical platform assembly

S2.1. Microchip preparation

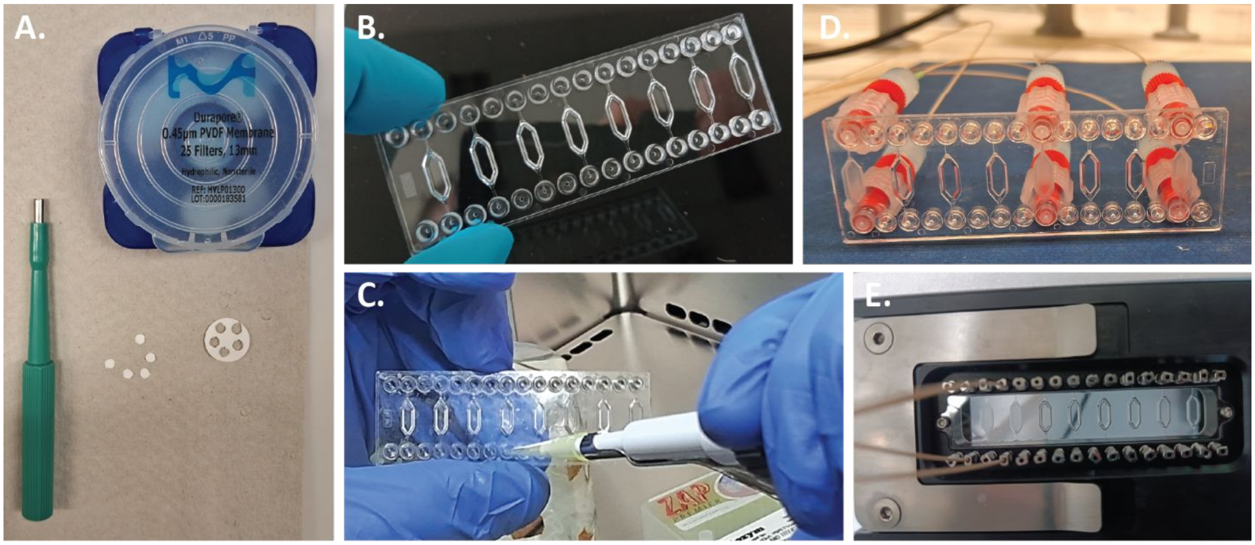

Figure S1. Overview of the steps to prepare the microchip for use: (A) PVDF filters are punched out with a biopsy punch; (B) 20 µL reaction chamber chip; (C) Loading the microchip with yeast inoculum; (D) Off-line use of the microchips with the filters placed in the outlet ports; (E) A microchip with two fermentations in the upper chambers, placed in the chip incubator.

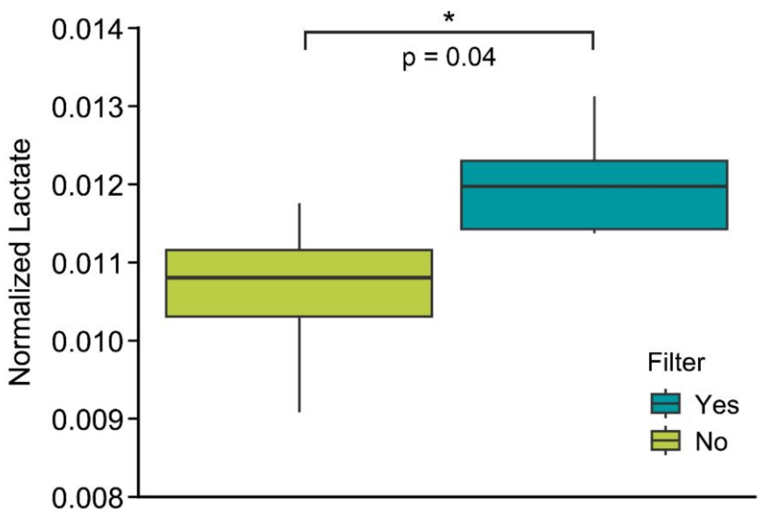

Figure S2. Data for lactate from off-line measurements of fermentation effluent across 20-hour fermentations from chambers with and without 0.45 µm PVDF filters placed in the outlet port. Lactate concentration (g/L) was normalized to starting OD of the inoculum. Higher lactate concentrations were measured from the fermentations with filters (N = 5). Statistical analysis comparing chips with and without filters was performed with a two-sided Welch Two Sample t-test with unequal variance. * represents a significance level of $p < 0.05$.

S2.2. Analytical platform components

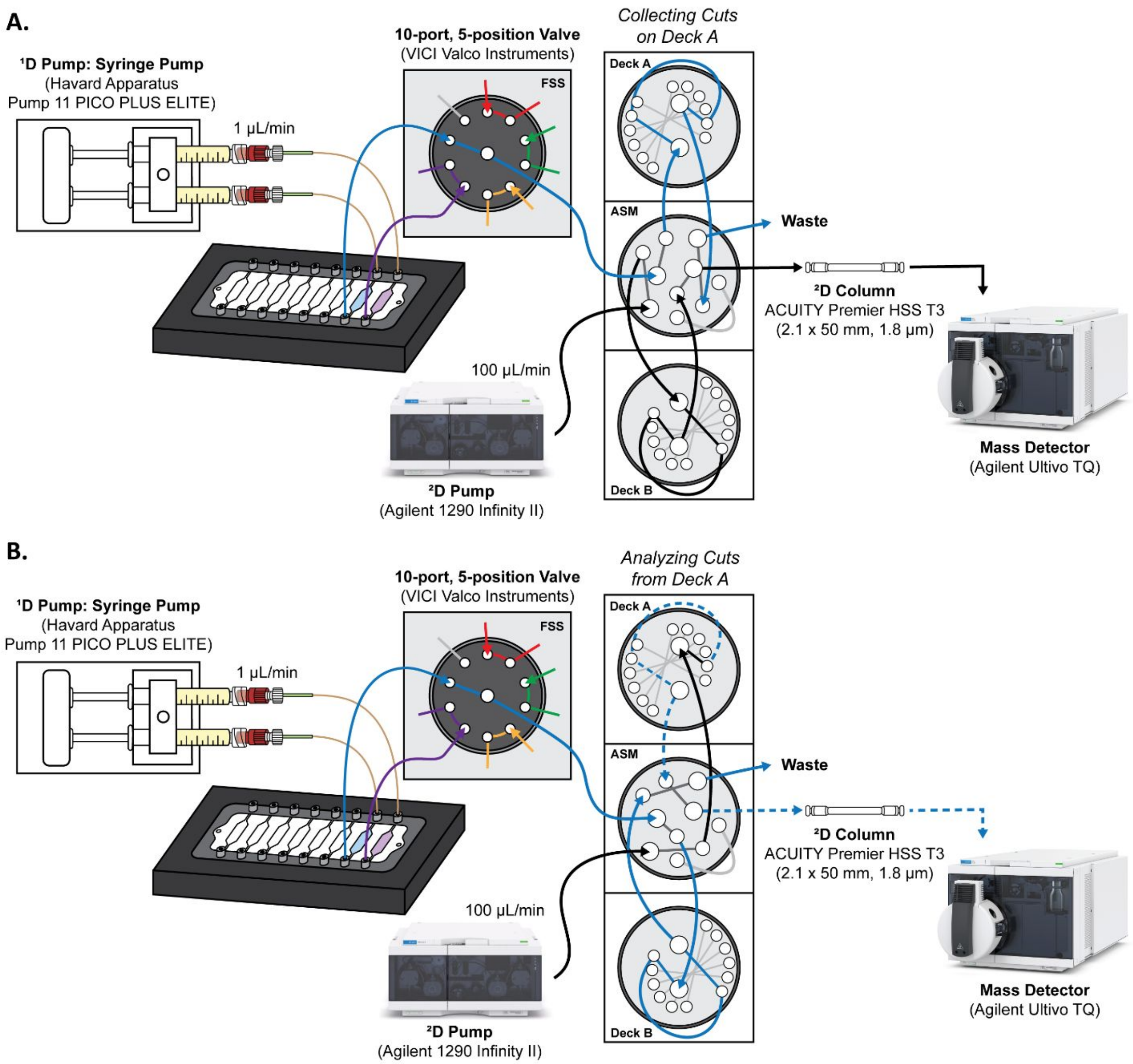

Figure S3. Detailed schematic representation of the entire 2DLC-MS/MS analytical µ-platform showing valve operation and flow paths in the 2D-LC hardware during (A) collection of the fermentation effluent fraction and (B) the analysis phase. The microchip inoculated with yeast cells is placed in the microchip incubator. The perfusion media is delivered at 1 µL/min via syringe pump. A second syringe pump is used for a 1 µL/min wash flow in between independent fermentations. The third syringe pump delivers a 10 µL/min wash flow at the end of each measurement. Switching of the multiplexed microchips is automatically done at specific timepoints by the UIB. Cuts are collected with 2D-LC hardware, undergo LC separation, and are analyzed by MS/MS. (Instrumentation images reproduced with permission courtesy of Agilent Technologies, Inc.)

Table S11. Details of the components in the multiplexed microchip fermentation interface.

FSS Valve (VICI Valco Instruments)	• 10-port, 5-position flow-through stream selector valve; 1/32" PEEK fittings, 0.25 mm bore size, PAEK stator and Valcon E rotor<br>• High-speed universal electric actuator; EUHA<br>• Mounting hardware; CMH13H
UIB (Agilent)	• Universal Interface Box II; G1390B<br>• General Purpose Cable GPIO-Open End; G1103-61611
Microchip (ChipShop)	• Reaction chamber chip; fluidic design 584, hydrophilized, Topas; 10001297<br>• Lab-on-a-Chip Cell Culture Incubator with heating elements

S3 Adapting the platform for microscale operation

S3.1. Hardware and setting modifications

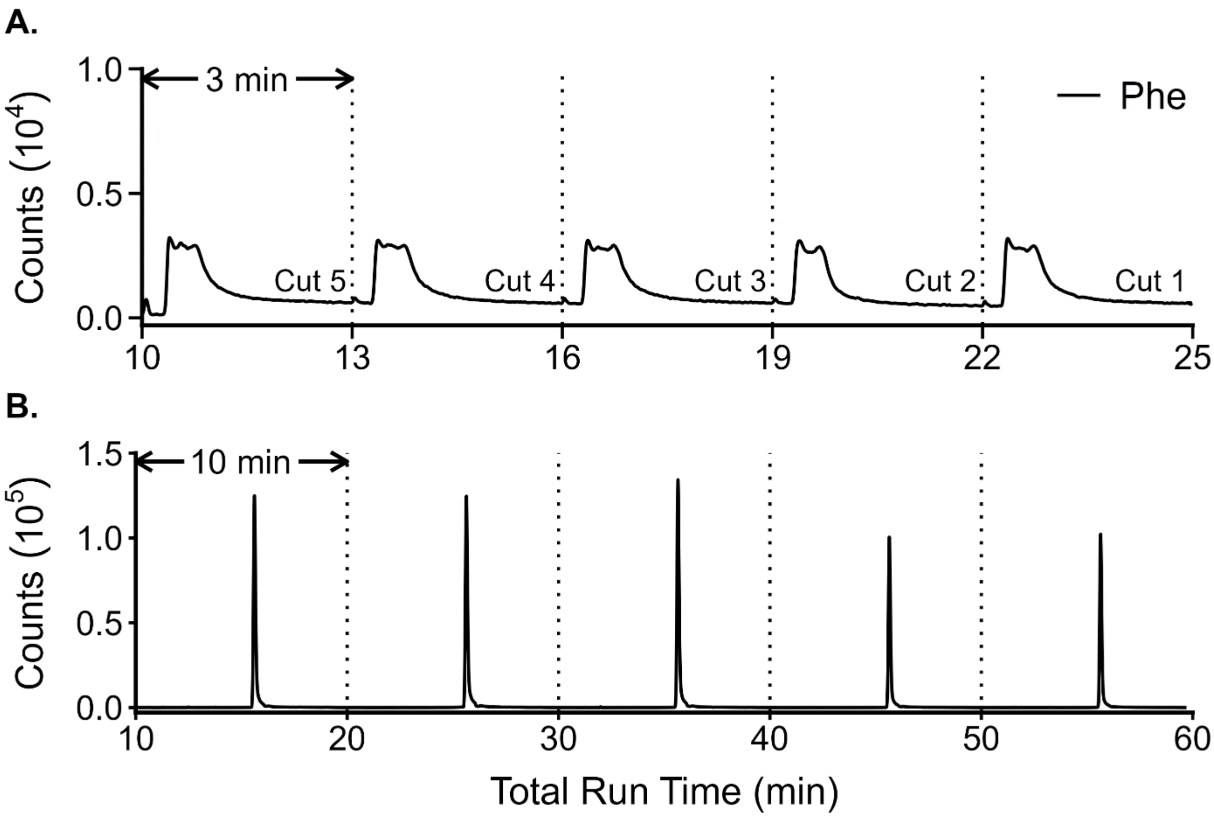

Figure S4. EICs of a solution containing 8 µmol/L phenylalanine measured using (A) a flow injection analysis without LC separation and (B) with LC column.

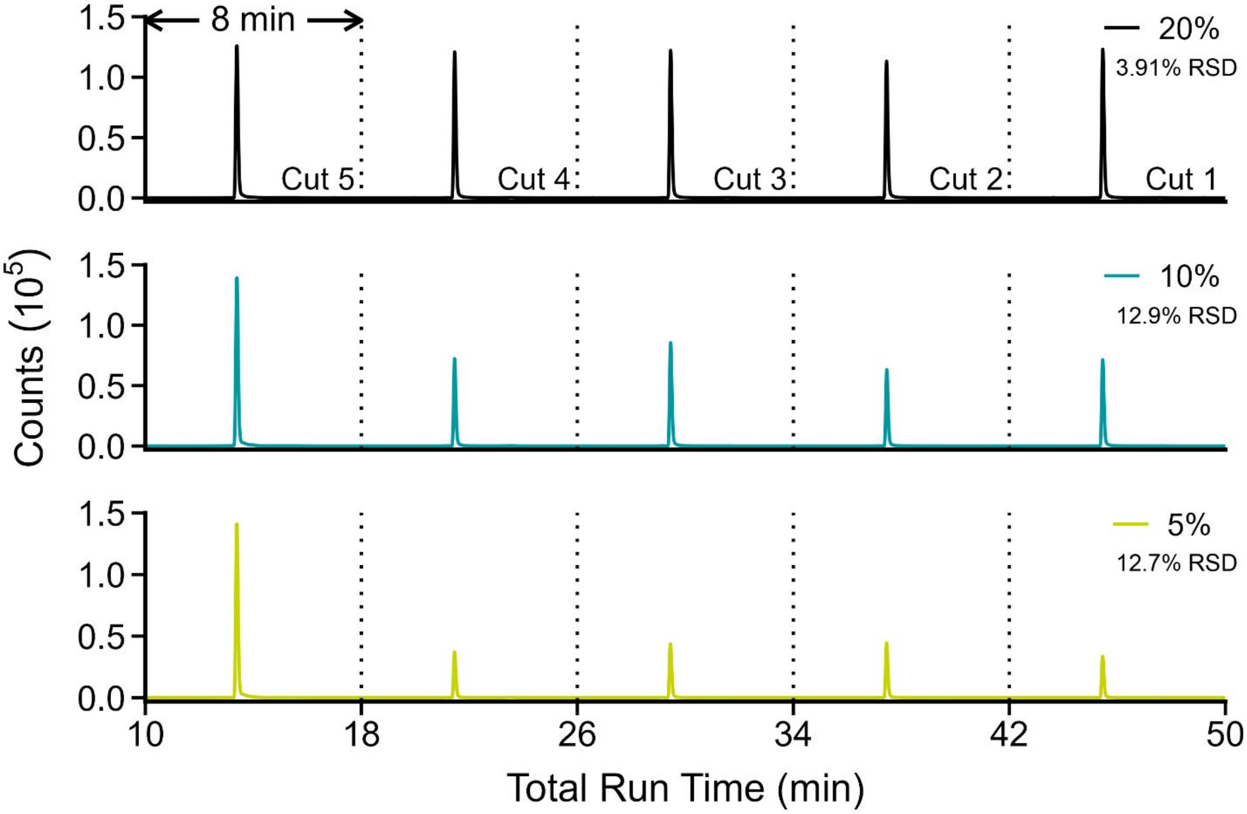

Figure S5. Comparison of 20%, 10%, and 5% sample loop filling in 10 µL sample loops with EICs of 8 µmol/L phenylalanine across five 2D-LC cuts with an 8-minute ^{2}D cycle time. Loop filling was increased by lowering the ^{1}D syringe pump flow rate from 1 µL/min to 250 nL/min. Peak area %RSD shown for each series.

S3.2. Flow path volumes

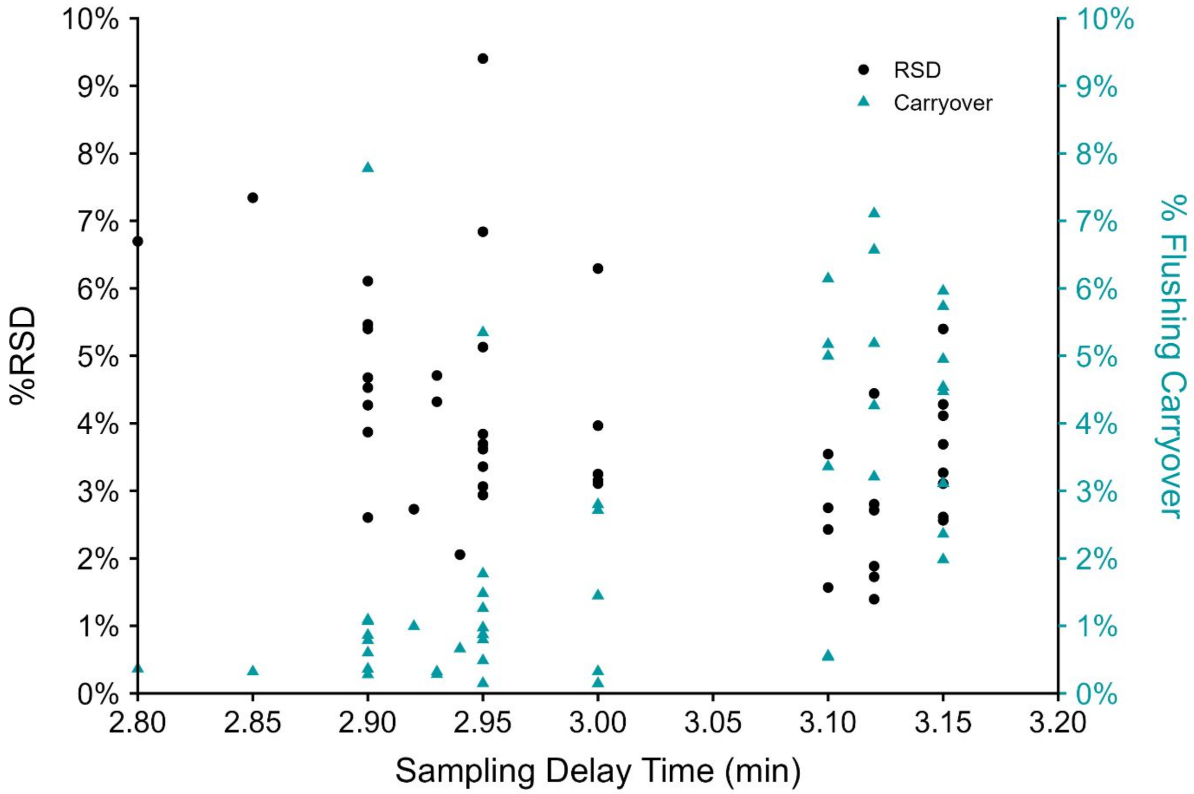

Figure S6. Experimental determination of the optimal sampling delay time based on the calculated volumes of the system. When prefilling the volume between the FFS valve to deck A with water, the first cut is affected with a reduced signal observed if the delay time is too short. While when prefilling this volume with sample, a peak from the flushing of the capillaries at the end of a measurement appears when the delay time is too long and the path is consequently overfilled. The sampling delay time should have a low flushing carryover signal and a low %RSD across the EIC peak areas from three collected cuts when prefilling with a solution containing 8 µmol/L phenylalanine. Based on the results, the optimal sampling delay time was selected as 2.90 min, an increase from the estimated sampling delay time by 0.38 min.

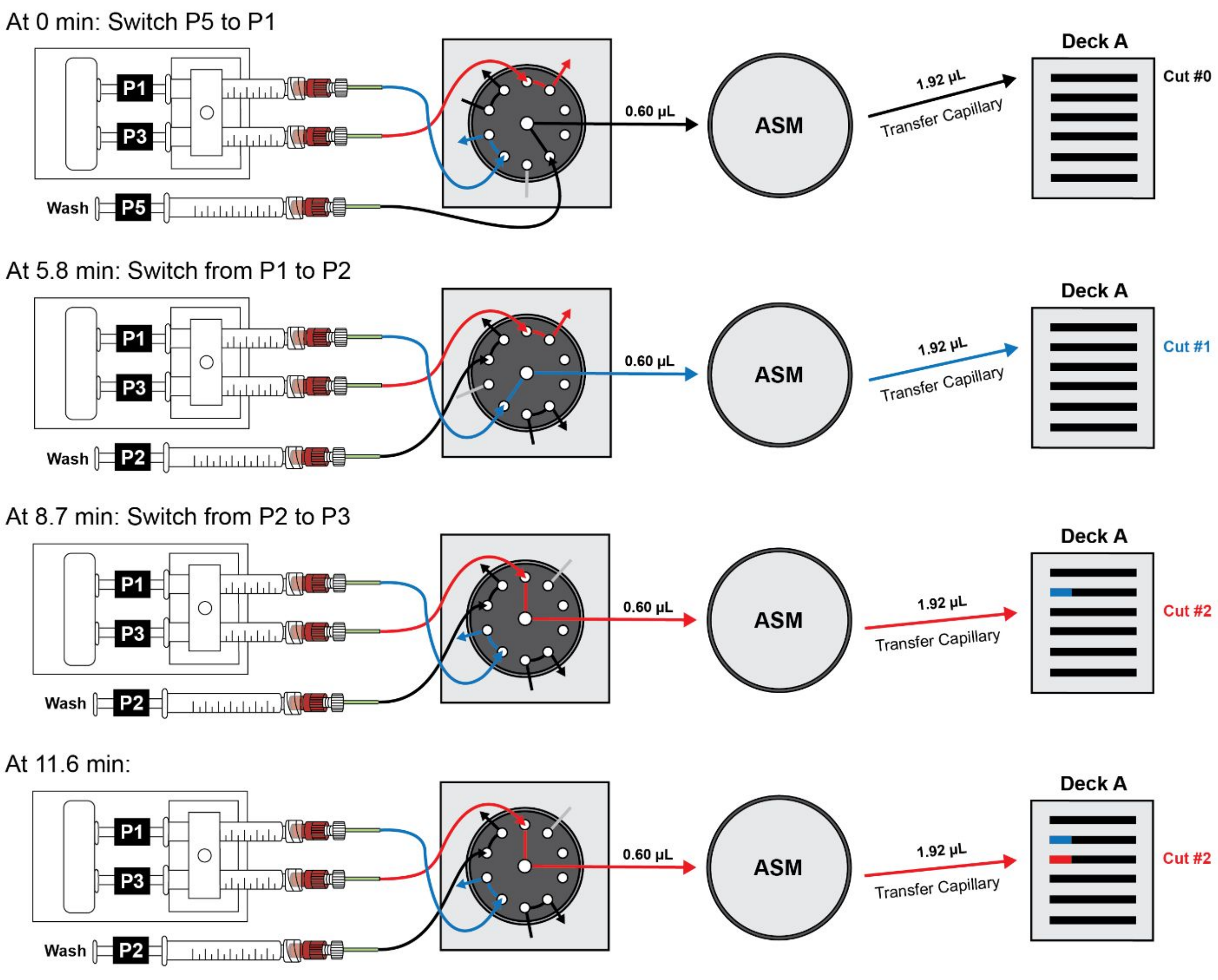

Figure S7. Schematic of the platform operating under optimal timing conditions with a sampling delay time of 2.9 minutes to match the flow path from the FSS valve to Deck A (0.60 µL + 1.92 µL + 0.38 µL). The time delay until collection of the cut begins, the sampling time, and the FSS valve switch timing are all set equal and a wash flow (P2) between independent fermentations (P1 and P3) is used to minimize contamination. A higher wash flowrate cleans the flow path after each measurement (P5).

S4 Monitoring metabolites from microscale fermentations

S4.1. Metabolites measured in single and multiplexed fermentations

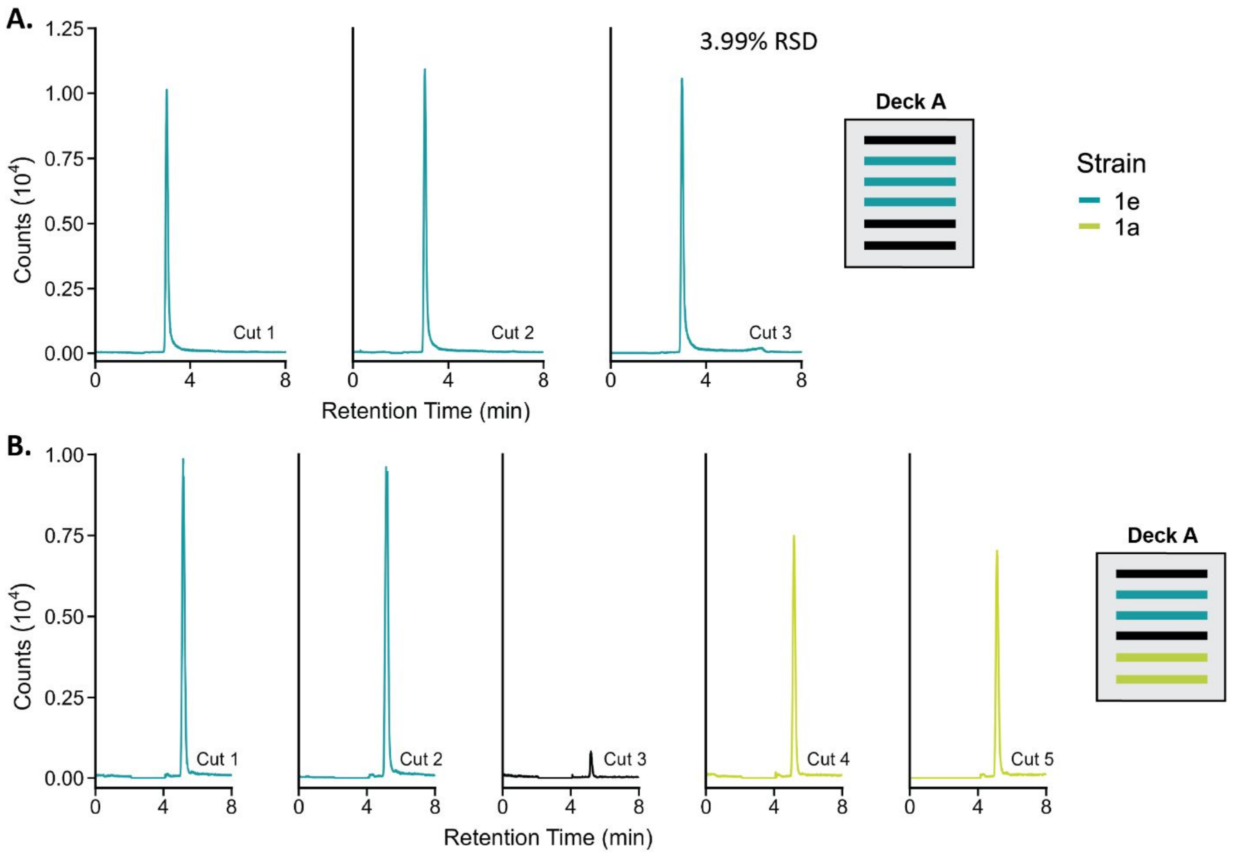

Figure S8. EICs of lactate produced by fermentations in which cuts were collected with an 8-minute ^{2}D cycle time. In (A) a single fermentation was monitored in a 20 µL reaction chamber chip 3.5 hours after inoculation and in (B) multiplexed fermentations were monitored with strains 1e and 1a. Cuts from the first fermentation were collected, and then after a wash step, cuts were collected from the second fermentation. Two different sample collection schemes were used on Deck A (see insets), allowing either monitoring of a single fermentation (A) within 40 minutes or two different fermentations to be monitored (with intermediate washing, B) within an hour.

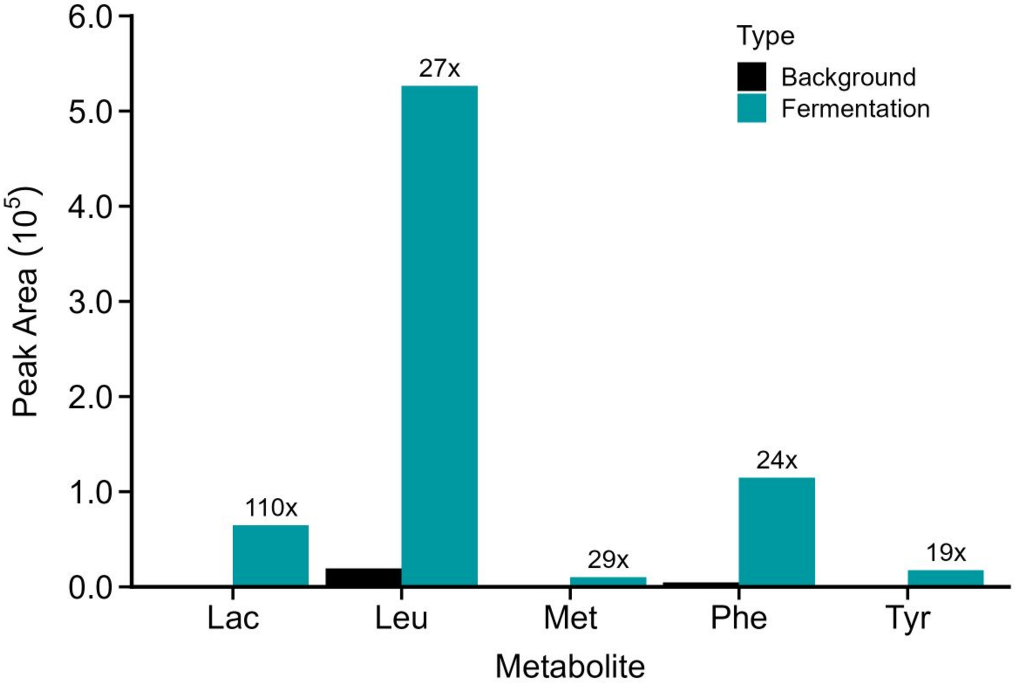

Figure S9. Background signals of metabolites in cell media compared to signals determined during a microscale fermentation of the lactate-producing 1e strain. The fold change (fermentation vs. media background) shown is for the peak area of the respective monitored metabolites.

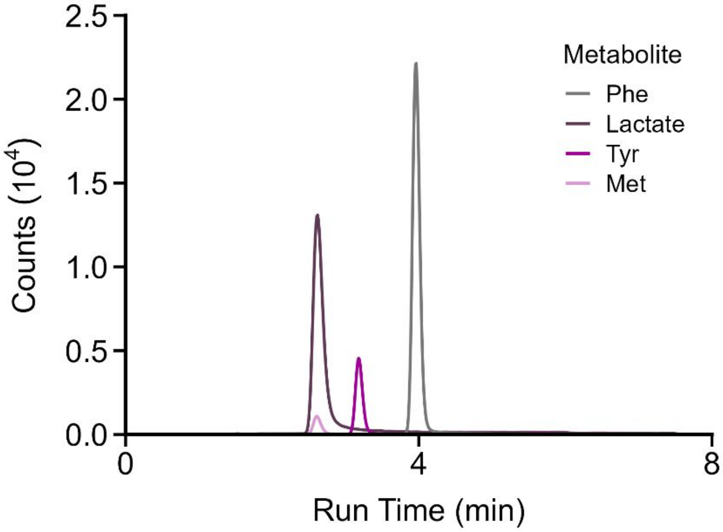

Figure S10. Representative EICs for metabolites (phenylalanine, lactate, tyrosine, methionine) monitored during an eight-hour microscale fermentation of the lactate-producing 1e strain.
